# Using Co-Expression Analysis and Stress-Based Screens to Uncover Arabidopsis Peroxisomal Proteins Involved in Drought Response

**DOI:** 10.1371/journal.pone.0137762

**Published:** 2015-09-14

**Authors:** Jiying Li, Jianping Hu

**Affiliations:** 1 Department of Energy Plant Research Laboratory, Michigan State University, East Lansing, Michigan, United States of America; 2 Genetics Graduate Program, Michigan State University, East Lansing, Michigan, United States of America; 3 Plant Biology Department, Michigan State University, East Lansing, Michigan, United States of America; Iowa State University, UNITED STATES

## Abstract

Peroxisomes are essential organelles that house a wide array of metabolic reactions important for plant growth and development. However, our knowledge regarding the role of peroxisomal proteins in various biological processes, including plant stress response, is still incomplete. Recent proteomic studies of plant peroxisomes significantly increased the number of known peroxisomal proteins and greatly facilitated the study of peroxisomes at the systems level. The objectives of this study were to determine whether genes that encode peroxisomal proteins with related functions are co-expressed in Arabidopsis and identify peroxisomal proteins involved in stress response using *in silico* analysis and mutant screens. Using microarray data from online databases, we performed hierarchical clustering analysis to generate a comprehensive view of transcript level changes for Arabidopsis peroxisomal genes during development and under abiotic and biotic stress conditions. Many genes involved in the same metabolic pathways exhibited co-expression, some genes known to be involved in stress response are regulated by the corresponding stress conditions, and function of some peroxisomal proteins could be predicted based on their co-expression pattern. Since drought caused expression changes to the highest number of genes that encode peroxisomal proteins, we subjected a subset of Arabidopsis peroxisomal mutants to a drought stress assay. Mutants of the LON2 protease and the photorespiratory enzyme hydroxypyruvate reductase 1 (HPR1) showed enhanced susceptibility to drought, suggesting the involvement of peroxisomal quality control and photorespiration in drought resistance. Our study provided a global view of how genes that encode peroxisomal proteins respond to developmental and environmental cues and began to reveal additional peroxisomal proteins involved in stress response, thus opening up new avenues to investigate the role of peroxisomes in plant adaptation to environmental stresses.

## Introduction

Peroxisomes are small and single membrane-delimited organelles that house numerous oxidative reactions connected to metabolism and development. These organelles are dynamic in nature, as their abundance, morphology and protein composition can be remodeled in response to developmental and environmental cues to adapt to the need of the organism [1,2,3]. Plant peroxisomes perform conserved functions such as β-oxidation of fatty acids and related metabolites and detoxification of reactive oxygen species (ROS), as well as plant-specific functions including photorespiration and metabolism of hormones such as jasmonate (JA) and auxin. Peroxisomes are crucial to virtually every developmental stage in plants, from embryogenesis, seedling development, vegetative and reproductive development, to senescence, and were recently shown to be involved in plant response to biotic and abiotic stresses [2,4]. The number of known peroxisomal proteins has risen to ~170 in Arabidopsis, largely due to recent peroxisomal proteome analyses followed by *in vivo* protein targeting verifications [5].

Peroxisomes possess many oxidative reactions that produce H_2_O_2_, as well as ROS-scavenging enzymes such as catalase and ascorbate-glutathione cycle enzymes [4,6]. ROS is a key component in stress responses [7]. Suppression of catalase 1 in tobacco resulted in necrotic lesions in high light and increased susceptibility to paraquat, salt and ozone [8]. Mutants of Arabidopsis catalase 2 develop photoperiod-dependent leaf lesions [9]. Evidence from melon, Arabidopsis and tobacco suggested the involvement of several peroxisomal photorespiratory enzymes, e.g., hydroxypyruvate reductase (HPR), serine:glyoxylate aminotransferase (SGT), alanine:glyoxylate aminotransferase (AGT), and glycolate oxidase (GOX) in immune response, possibly through ROS production [10,11,12].

Peroxisomes are also involved in stress response through mechanisms other than ROS homeostasis. Arabidopsis Ca^2+^-dependent protein kinase CPK1 is physically associated with peroxisomes and functions in a SA-dependent signaling pathway that leads to plant resistance to both fungal and bacterial pathogens [13,14]. Arabidopsis PEN2 is a peroxisome-associated myrosinase involved in callose deposition and glucosinolate hydrolysis necessary to generate antimicrobial products, thus is required for plant resistance against a broad spectrum of nonhost fungal pathogens, [15,16,17,18,19]. Furthermore, JA biosynthetic enzymes, some of which reside in peroxisomes, have been shown to affect systemic acquired resistance (SAR) to varying degrees [20]. It was suggested that the final step of SA biosynthesis, i.e., cinnamate to SA via the reduction of two carbons, may occur through β–oxidation in the peroxisome [21], thus making the peroxisome a potential player in SAR signaling. Interestingly, some virus species can hijack peroxisomes for viral RNA replication, causing the proliferation of peroxisome-like vesicle structures and leading to plant necrosis [22,23], which adds another layer of peroxisomal involvement in plant-pathogen interaction.

Despite these findings, there are still substantial knowledge gaps in the role of peroxisomes in stress response and how the functions of these peroxisomal proteins may be connected. To further identify peroxisomal proteins involved in plant response to various stress conditions, a peroxisome-centered systematic approach is needed. Recent advances in genome-wide transcriptomic and gene ontology enrichment analyses have provided valuable information on gene functions and mechanisms of biological processes. An important finding from these analyses is that genes functioning in the same pathway are often co-regulated by shared transcriptional regulatory systems and thus co-express across development and/or under many stress conditions [24]. To this end, we performed a genome-wide transcriptomic analysis of genes that encode peroxisomal proteins in Arabidopsis, trying to determine whether peroxisomal genes involved in the same biochemical pathways are co-expressed and whether we could identify new peroxisomal proteins involved in stress response using this type of *in silico* analysis. We followed up the *in silico* analysis with a pilot drought-based mutant screen, which identified the role of the peroxisomal LON2 protease and the photorespiratory enzyme hydroxypyruvate reductase 1 (HPR1) in drought resistance. Our study marks the beginning of systematic identifications of peroxisomal proteins involved in plant adaptation to stresses.

## Materials and Methods

### Plant materials and growth conditions

*Arabidopsis thaliana* ecotype Col-0 was used as wild type (WT). T-DNA insertion mutant lines were obtained from the Arabidopsis Biological Resource Center (ABRC; http://www.arabidopsis.org/) and confirmed by PCR genotyping. Seeds were sown in the soil, stratified in the dark at 4°C for 3 days, and plants were grown in a controlled growth chamber at 22°C under long-day conditions (16 hrs white light at 100 μmol photons m^-2^ s^-1^ and 8 hrs dark) for 3.5 weeks before drought treatment.

### Microarray data analysis and heatmap visualization

Microarray datasets containing expression data of Arabidopsis peroxisomal genes from various tissues at different developmental stages were obtained from the AtGenExpress database, and expression data under biotic and abiotic stresses were downloaded from NCBI Gene Expression Omnibus (GEO) database (Table A in S1 File). Peroxisomal gene expression data obtained from various developmental stages were directly extracted from the whole-genome data and used for generating the heatmap. For data on biotic and abiotic stresses, log2-normalized data were extracted for peroxisomal genes from the whole-genome expression profile, using methods previously described [25]. Analysis was performed using the Bioconductor software [26] with the statistical computing language R (version 2.15.2). Normalization of gene expression values was carried out with the robust multi-array average (RMA) algorithm [27] implemented in the Affy package of Bioconductor. Statistical significance of the differential expression values were assessed with Linear models for microarray (limma) package [28]. Hierarchical clustering of the differentially expressed genes was visualized by creating heatmaps using the color palette package RColorBrewer and the gplots package [29].

### Chlorophyll fluorescence measurements

Chlorophyll fluorescence images of intact plants were obtained from a custom-designed plant imager chamber, using a previously described method [30]. Plants in the pots were placed in the imaging chamber in the dark for 20 min for dark adaptation before minimal chlorophyll fluorescence F_o_ was measured. Later, maximal fluorescence F_m_ was measured when a saturating pulse of light was applied. F_v_/F_m_ = (F_m_-F_o_)/F_m_. Fluorescence images were analyzed by ImageJ [31].

### Drought stress assays

For the drought tolerance screen, each selected mutant (two plants) and two WT plants were grown in the same pot under long day conditions (specified above) for 3.5 weeks, after which point plants stopped receiving water for 18 days before F_v_/F_m_ measurement was conducted. For the follow-up analysis of the *lon2* and *hpr1* mutants, F_v_/F_m_ measurement was repeated in the same way as in the primary screen, and watered plants were added as the control. Leaf samples from the drought-treated and control plants were harvested for chlorophyll content measurement, relative water content (RWC) and anthocyanin quantification as described previously [32].

For chlorophyll measurement, rosette leaves were weighed and placed into 2 ml 80% acetone in the dark for 3 days. Absorbance at 645 nm and 663 nm was measured using a spectrophotometer. Total chlorophyll content = (22.22 x A_645_ + 9.05 x A_663_) μg/ml x 2 ml /leaf fresh weight in mg.

To measure relative water content, rosette leaves were cut and immediately weighed as fresh weight (FW), and then placed in distilled deionized water at 4°C in the dark for 24 hrs, and the weight was recorded as turgid weight (TW). Then the rosette leaf sample was placed at 60°C for 2 days and the weight was recorded as dry weight (DW). Relative water content = (FW-DW) / (TW-DW) X 100%.

For anthocyanin measurement, rosette leaves were weighed, frozen by liquid nitrogen, and ground to powder. After adding 2 ml extraction buffer (1% HCl in methanol), the samples were placed at 4°C overnight. Later, an equal amount of chloroform was added, and the mixture was centrifuged for 5 min. After the top supernatant was transferred to a new tube, equal volume of 60% extraction buffer was added. Absorbance of each tube at 530 nm and 657 nm were measured with a spectrophotometer. Anthocyanin content = (A_530_-A_657_) /weight.

## Results

### Co-expression analysis of genes that encode peroxisomal proteins during development and in response to stresses

Microarray datasets containing expression data of Arabidopsis peroxisomal genes from various tissues at different developmental stages and under biotic and abiotic stresses were downloaded from the AtGenExpress database and NCBI Gene Expression Omnibus (GEO) database, respectively (Table A in S1 File). Developmental data were obtained from different tissues from seedlings, adult and senescing leaves, flowers, and siliques and seeds at various maturation stages. Abiotic stress conditions included high light, cold, hypoxia, drought, salt and the major stress hormone abscisic acid (ABA). Biotic stresses included the bacterial pathogen *Pseudomonas syringae* pv. *Tomato* (*pst*) DC3000, fungal pathogen *Botrytis cinerea*, and Pathogen-Associated Molecular Patterns (PAMPs) such as the bacterial flagellin 22 (flg22), bacterial elongation factor (elf18) and the fungal elicitor chitin (Table A in S1 File). Expression profiles of 160 peroxisomal genes (Table B in S1 File) were extracted from the whole-genome expression profile, clustered by hierarchical clustering analysis based on the extent of co-expression, and visualized by heatmaps.

**Fig 1:**
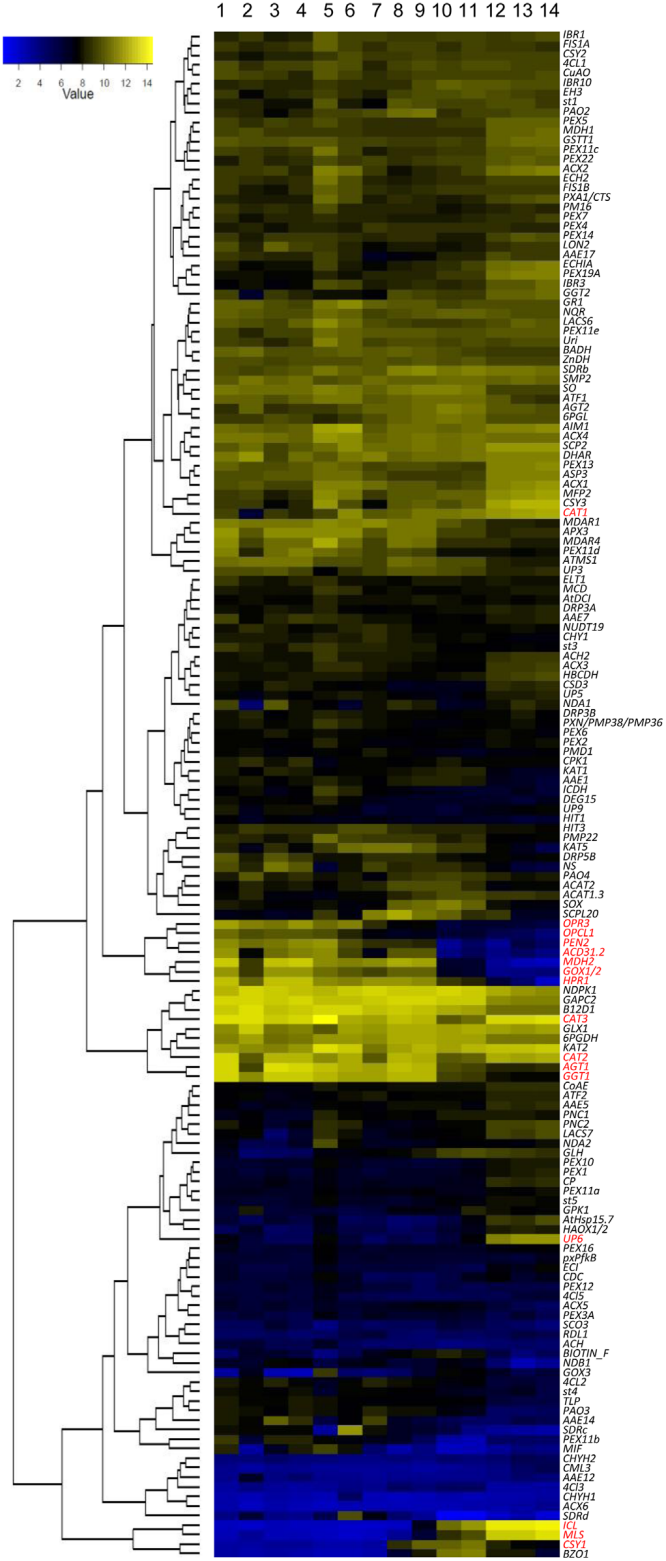
Heatmap of transcript levels of peroxisomal genes in various developmental stages. Absolute gene expression values downloaded from the AtGenExpress database were used for heatmap generation. Genes discussed in the text are in red. Developmental stages include: 1. seedling_cotyledons; 2. seedling_hypocotyl; 3. seedling_leaves1+2; 4. adult_leaves; 5. senescing leaves; 6. flower; 7. silique_stage3; 8. silique_stage4; 9. silique_stage5; 10. seed_stage6; 11. seed_stage7; 12. seed_stage8; 13. seed_stage9; 14. seed_stage10.

Not surprisingly, many peroxisomal genes that function in the same metabolic pathways are co-regulated during development (Fig 1). For example, genes that encode glyoxylate cycle enzymes isocitrate lyase (ICL), malate synthase (MLS), and citrate synthase 1 (CSY1) are clustered together and co-up-regulated during seed maturation but co-repressed in other developmental stages (Fig 1). This is consistent with the fact that the glyoxylate cycle, a pathway that converts the β–oxidation product acetyl-CoA to succinate and malate to be used for gluconeogenesis, is primarily if not exclusively active in seeds and early seedling development [2,33]. In agreement with their roles in photorespiration, which recycles 2-phosphoglycolate produced by the oxygenase activity of RuBisCO back to the Calvin-Benson cycle [34], the expression of genes for the peroxisomal photorespiratory enzymes hydroxypyruvate reductase 1 (HPR1), glycolate oxidase 1 and 2 (GOX1 and GOX2, which are indistinguishable in microarrays due to high sequence identity), and peroxisomal malate dehydrogenase MDH2 is high in vegetative tissues but diminished during seed development. In contrast to these three clustered genes, genes that encode two other photorespiratory enzymes, glutamate: glyoxylate aminotransferase 1 (GGT1) and alanine: glyoxylate aminotransferase 1 (AGT1), are expressed during seed development as well, indicating that photorespiration may not be the only process that these two enzymes participate. Interestingly, genes that encode the JA biosynthetic enzymes 12-oxophytodienoate reductase 3 (OPR3) and OPC-8:0 CoA ligase 1 (OPCL1), disease related protein PEN2, and the small heat shock protein ACD31.2 are clustered together and also co-expressed with the photorespiration genes *HPR1*, *GOX1/2* and *MDH2* throughout development. This pattern indicates that these stress-related and development-related (in the case of JA biosynthesis) genes might be under similar regulatory circuitry as those photorespiration genes. As the major H_2_O_2_ detoxification enzymes, the three catalases are mostly constitutively expressed throughout development (Fig 1).

**Fig 2:**
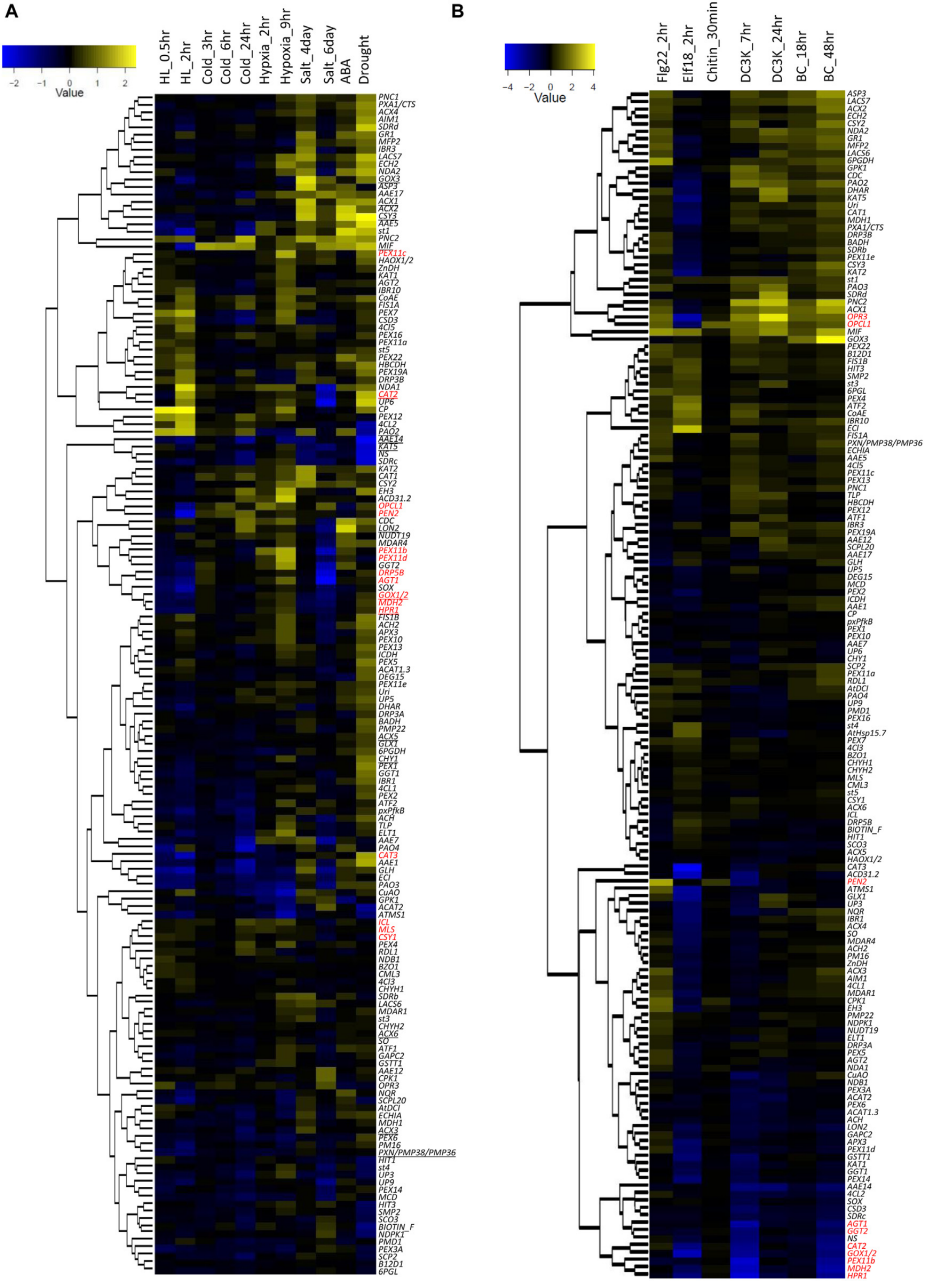
Heatmaps showing peroxisomal gene expression under stress conditions. Expression values are log2 normalized fold changes from untreated plants. Genes discussed in the text are in red. (A) Gene expression under abiotic stresses. Genes subjected to mutant analysis are underscored. (B) Gene expression under biotic stresses.

Transcriptional reprogramming during stresses is an important mechanism to confer stress tolerance [35,36]. Many genes that encode peroxisomal proteins are significantly regulated by abiotic stresses (Fig 2A). Among them, the small heat shock protein-encoding gene *AtHsp15*.*7* showed >150-fold increase in expression under high light (Figure A in S1 File) and had to be removed from the heatmap in Fig 2A to prevent it from masking the changes in other genes in the heatmap. Genes encoding the peroxisomal proliferation factors PEX11b, PEX11c and PEX11d are all up-regulated by hypoxia (Fig 2A), which is consistent with a previous finding that hypoxia stress can rapidly stimulate peroxisomal extension over endoplasmic reticulum [37]. *CAT2* and *CAT3* expressions are also significantly up-regulated during drought stress (Fig 2A), consistent with their role as major ROS detoxification enzymes in stress response [38]. The glyoxylate cycle genes *ICL*, *MLS* and *CSY1* are again clustered (Fig 2A), suggesting the tight regulation of this pathway by abiotic stress factors. Genes encoding the photorespiration enzymes GOX1/2, HPR1, MDH2, AGT1 and the chloroplast/peroxisome dual localized organelle division protein dynamic related protein 5B (DRP5B) are clustered (Fig 2A), raising the interesting possibility that photorespiration and the proliferation of peroxisomes are co-regulated during plant adaptation to abiotic stresses.

In response to biotic stresses, genes for some peroxisomal proteins previously shown to be involved in defense exhibited strong transcriptional reprogramming. For example, *PEN2* (*Penetration 2*) is induced by two PAMPs, flg22 and chitin, but not by elf18 or the two pathogens (Fig 2B), supporting its major role as a myrosinase in PAMP-triggered immunity [15,16]. The two JA biosynthetic genes *OPR3* and *OPCL1* are co-up-regulated by flg22, chitin, *P*. *syringae* and *B*. *cinerea* (Fig 2B), consistent with JA’s role as an important defense hormone [39]. Interestingly, photorespiratory genes such as *HPR1*, *CAT2*, *GOX1/2*, *MDH2*, *AGT1* and *GGT2* are co-down-regulated by elf18, *P*. *syringae* and *B*. *cinerea* (Fig 2B), which is in agreement with the idea that photorespiration may play a defense role against pathogens through H_2_O_2_-dependent and -independent metabolisms [40]. The peroxisomal elongation factor gene *PEX11b* is again co-expressed with several photorespiratory genes during biotic stresses (Fig 2B), which is in accordance with its co-expression with photorespiratory genes in response to light [41] and the role of PEX11b in inducing peroxisomal proliferation during dark-to-light transition [42]. This data also indicated a potential need to increase peroxisomal abundance during pathogen defense.

### A drought tolerance mutant screen revealed the role of the LON2 protease and the photorespiratory enzyme hydroxypyruvate reductase 1 (HPR1) in drought response

Based on the rule of “guilt-by-association” [24], those peroxisomal genes that showed significant up-regulation of transcript levels by some stresses have the potential to play a role in these specific conditions. To test this hypothesis, we decided to choose a stress condition, under which significant regulation of expression is seen for the highest number of peroxisomal genes, to screen for mutants with altered response.

Among the abiotic and biotic stress conditions examined, drought and the bacterial PAMP elf18 trigger expression changes to the highest number of peroxisomal genes (Figure B in S1 File). Drought is one of the most common environmental stresses that limit plant growth and development. Plants have evolved sophisticated adaptive drought tolerance mechanisms, including increased level of water transporting capacity, decrease of evaporative water, up-regulation of osmolytes and chaperone proteins, activation of Ca^2+^-dependent, ABA-dependent and other signaling pathways, and regulation of the transcript levels of the genes involved [43]. Mutant plants defective in these processes may exhibit increased drought sensitivity, such as increased water loss and ion leakage, decrease of photosynthesis rate, degradation of chlorophyll, and eventually cell death and plant withering [43]. As such, we used a drought tolerance assay as an initial screen to test the prediction from *in silico* analysis.

We have a collection of Arabidopsis mutants, which has facilitated us in discovering functions of newly identified peroxisomal proteins in previous studies [25,44,45]. To identify peroxisomal proteins involved in drought stress response, we first selected 26 mutants for 18 genes, most of which showed transcript level changes under drought or the drought stress hormone ABA. These included the up-regulated genes *CAT2*, *GOX3*, *Hsp15*.*7*, *CSY3*, *Macrophage Migration Inhibitory Factor 1* (*MIF1*) and *LON2* protease, and the down-regulated genes polyamine oxidase *PAO2*, thiolase *KAT5* and acyl-CoA activating enzyme *AAE14*. We also included mutants of proteins involved in major peroxisomal pathways (photorespiration and fatty acids β-oxidation) but do not show obvious changes in transcript levels under drought, i.e. *hpr1*, *gox1*, *acx3*, *acx6*, and *acx1 acx5*. Mutants of mildly regulated genes, such as peroxisomal NAD^+^ transporter *PXN* and beta-hydroxyisobutyryl-CoA hydrolase *CHY1* were assayed as well (Table C in S1 File).

**Fig 3:**
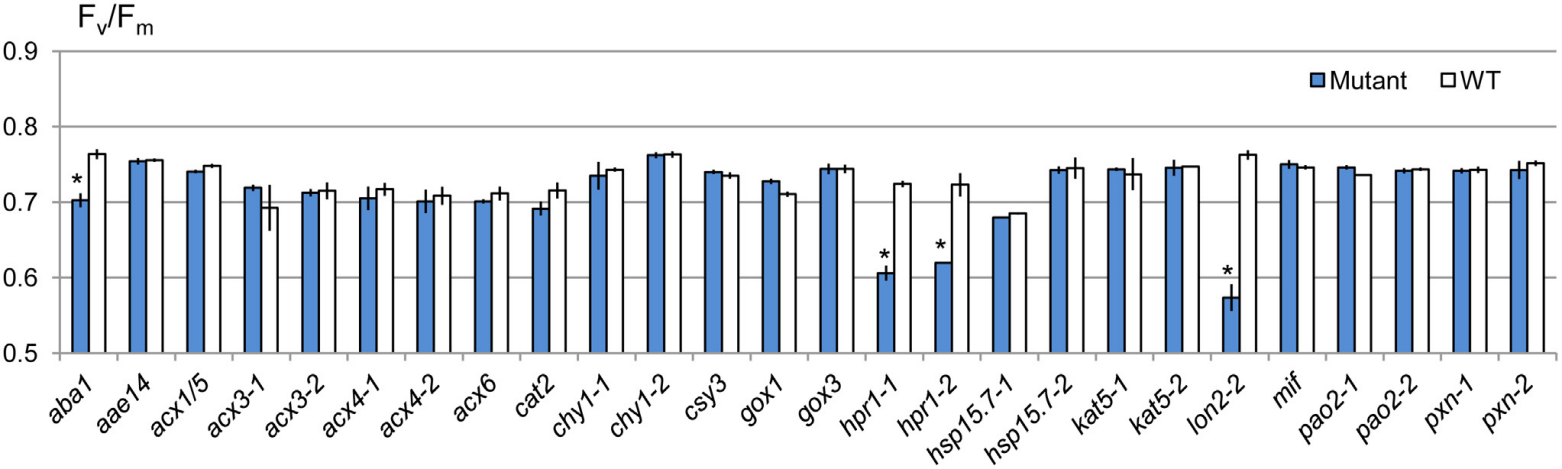
F_v_/F_m_ comparison between the selected peroxisomal mutants and wild type plants grown in the same pot. The *aba1* mutant (salk_059469) was used as a positive control. Two biological replicates were used for each genotype. Asterisk indicates p-value < 0.01 in Student’s *t* test.

For an efficient and quantitative drought tolerance assessment, we used the photosynthetic efficiency F_v_/F_m_ as a drought susceptibility indicator in our screen. Each mutant was grown in the same pot with the wild type plant for 3.5 weeks with periodic irrigation, followed by an 18-day drought period, at the end of which F_v_/F_m_ was measured. The positive control, ABA biosynthetic mutant *aba1* [46], and mutants for the LON2 protease and photorespiratory enzyme HPR1, showed statistically significant decrease in F_v_/F_m_ after drought treatment (Fig 3).

**Fig 4:**
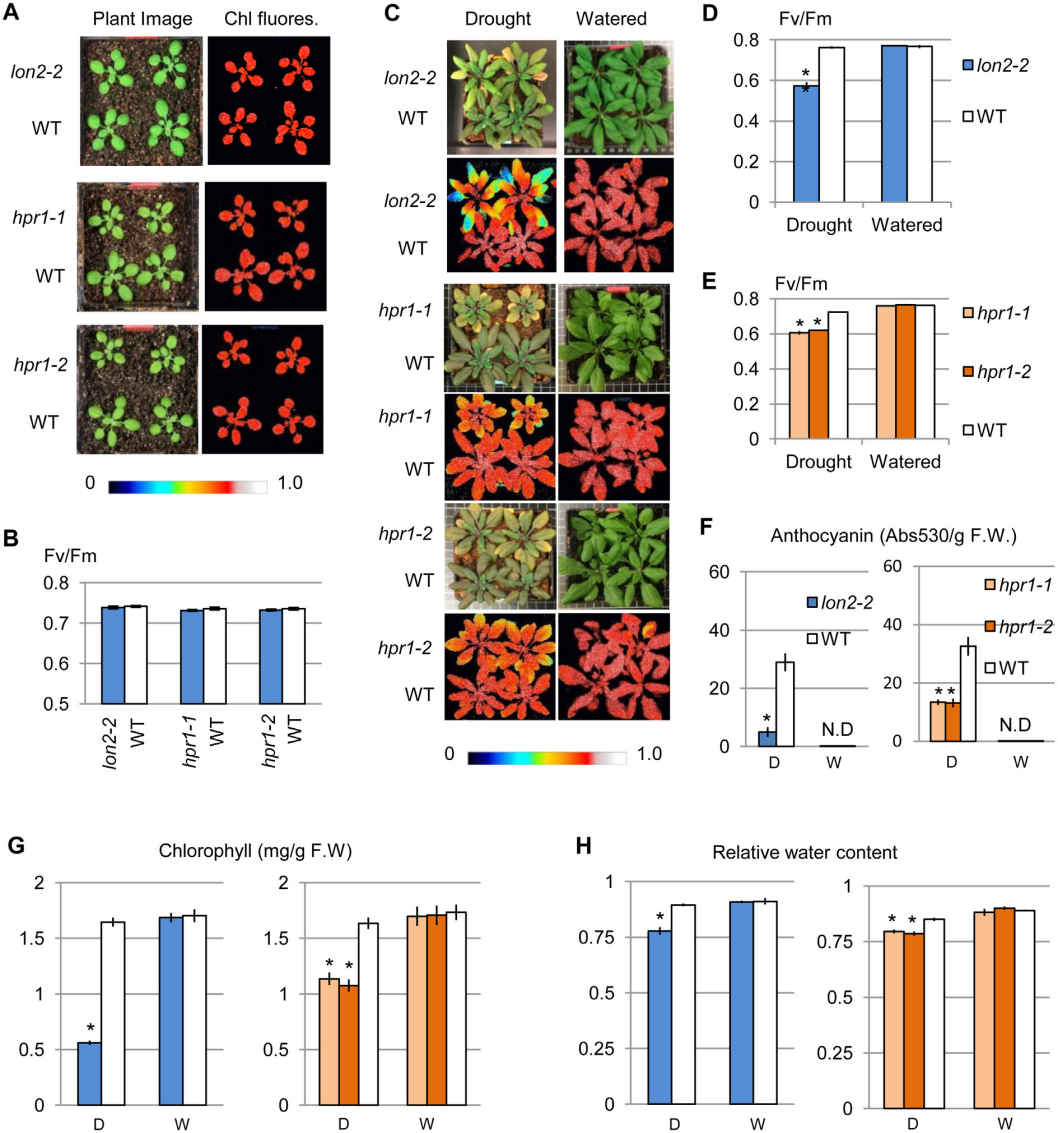
Drought resistance phenotypes of *lon2* and *hpr1* mutants. (A) Images of plants (left) and color-coded chlorophyll fluorescence that indicates F_v_/F_m_ values (right). (B) F_v_/F_m_ comparison between mutants and wild type. Four biological replicates of each genotype were used. No significant difference in F_v_/F_m_ was observed. (C) Plant images and color-coded chlorophyll fluorescence images that indicate F_v_/F_m_ values. (D-E) F_v_/F_m_ comparison between mutants and wild type. Two biological replicates were used for each genotype under each condition. Asterisk, p < 0.01 in Students’ *t* test. (F-H) Quantification of anthocyanin (F), chlorophyll (G), and relative water content (H) in mutants and wild type plants. Three biological replicates were used for each genotype under each condition. Asterisk indicates p < 0.01 in Students’ *t* test; N.D, not detectable; D, drought; W, watered.

The *lon2* and *hpr1* mutants were further analyzed to assess defects in drought resistance. Prior to drought treatment, *lon2* and *hpr1* mutants exhibited similar F_v_/F_m_ values to that of the wild type (Fig 4A and 4B), suggesting that the drought sensitive phenotypes we observed were specific to drought stress and not a result from general growth defect. Compared with watered plants and drought-treated wild type plants, drought-treated *lon2-2*, *hpr1-1* and *hpr1-2* displayed an age-dependent gradient of photosynthetic defect, which was stronger in older leaves and milder in young leaves (Fig 4C and 4D). These mutants also showed defects in other drought stress indicators, including reduced anthocyanin induction (Fig 4F), accelerated chlorophyll degradation (Fig 4G), and lower relative water content (Fig 4H).

## Discussion

We have constructed peroxisome-centered transcriptomic heatmaps using Arabidopsis microarray data from various developmental stages and under biotic and abiotic stresses. Results obtained from the *in silico* analysis not only showed correlation between protein function and expression regulation for many proteins with known function, but also provided information from which previously unknown roles may be inferred for some peroxisomal proteins. For example, out of the two peroxisomal *MDH* isoforms, *MDH2* is tightly co-expressed with *HPR1* and *GOX1/2*, suggesting that MDH2 is the major MDH isoform that functions in photorespiration. Among all the genes analyzed, *AtHsp15*.*7*, shows the strongest up-regulation by high light, suggesting that the small heat shock protein Hsp15.7, which was shown to be a stress-inducible constituent of the peroxisome [47], may facilitate the re-folding of proteins that have been partially unfolded or damaged under high light stress. Up-regulation of peroxisome elongation factors such as *PEX11b*, *PEX11c* and *PEX11d* under hypoxial and biotic stresses suggests that increased volume of peroxisome might be a mechanism for the plant to deal with enhanced oxidative stress. Finally, the fact that unknown protein UP6, which was previously shown to play a minor role in β–oxidation [45,48,49], is strongly up-regulated during late seed developmental stages, indicates a possible role for this protein in seed maturation.

Following the transcriptome analysis, we used a drought stress assay to test promising gene candidates, and identified LON2 and HPR1 as contributors to drought tolerance. HPR1 converts hydroxypyruvate to glycerate during photorespiration [2]. It is possible that drought induces stomatal closure, which limits the atmospheric uptake of CO_2_, thus activating the oxygenase activity of RuBisCO and subsequently, photorespiration. In *hpr1*, the accumulated photorespiratory metabolites may inhibit RuBisCO activity and slow down the Calvin-Benson cycle due to decreased supply of glycerate, thus leading to accumulated NADPH and ROS that cause a series of oxidative damages. Mutants for the other two genes directly (*GOX1*) or indirectly (*CAT2*) involved in photorespiration did not show a drought phenotype, possibly due to their functional redundancy with *GOX2* and *CAT1/CAT3*, respectively.

LON2 is a protease with unknown substrates and a role in peroxisomal matrix protein import and degradation [50,51]. Our transcriptomic analysis found *LON2* to be up-regulated by ABA by 4-fold, which is consistent with its 8-fold induction by ABA in guard cells [52], suggesting that LON2 may play a role in drought response through ABA signaling and peroxisomal protein quality control pathways. Since peroxisomal degradation via autophagy was shown to be enhanced in the *lon2* mutant, especially in older leaves [50,53], it is possible that there are insufficient peroxisomes in the *lon2* mutant to carry out photorespiration, which is critical for plant survival under drought conditions. This may also explain why *lon2* and *hpr1* display stronger phenotypes than the ABA biosynthetic mutant *aba1* in the initial F_v_/F_m_ screen, because our screen measured photosynthetic efficiency, which is directly impacted by photorespiration deficiencies in these two peroxisomal mutants.

Although *in silico* analysis is powerful for function prediction, mutants for many genes whose transcript levels are regulated by drought did not exhibit obvious drought tolerance defects. Given the manageable size of the peroxisomal proteome and available mutants, stress-based mutant screens should be a more direct way to identify peroxisomal proteins involved in stress response. In this initial screen, we identified strong drought sensitive phenotypes in the knockout mutants of the LON2 protease and the photorespiratory enzyme HPR1, suggesting that future larger-scale screens would be promising to investigate the role of peroxisomes in plant adaptation to environmental stresses comprehensively. The next step would be to link these peroxisomal proteins with the global stress response networks.

## Supporting Information

S1 File Supporting information. This file includes the following: **Figure A**. **Expression of the small heat shock protein gene *AtHsp15*.*7* in response to abiotic stresses; Figure B**. Total number of Arabidopsis peroxisomal genes with significantly changed expression levels in response to stresses; **Table A**. Microarray datasets used in this study; **Table B**. Arabidopsis peroxisomal gene list; **Table C**. Mutants used in the primary screen for drought tolerance.
(PDF)
