# Supplementary material for: Using Co-Expression Analysis and Stress-Based Screens to Uncover Arabidopsis Peroxisomal Proteins Involved in Drought Response
Source: PLoS One. 2015 Sep 14;10(9):e0137762. doi: 10.1371/journal.pone.0137762 (PMC4569587; doi:10.1371/journal.pone.0137762)
Supplement: S1 File — This file includes the following: Figure A. Expression of the small heat shock protein gene AtHsp15.7 in response to abiotic stresses; Figure B. Total number of Arabidopsis peroxisomal genes with significantly changed expression levels in response to stresses; Table A. Microarray datasets used in this study; Table B. Arabidopsis peroxisomal gene list; Table C. Mutants used in the primary screen for drought tolerance. (PDF) [file pone.0137762.s001.pdf]

This S1 File contains the following:

Fig A
Fig B
Table A
Table B
Table C

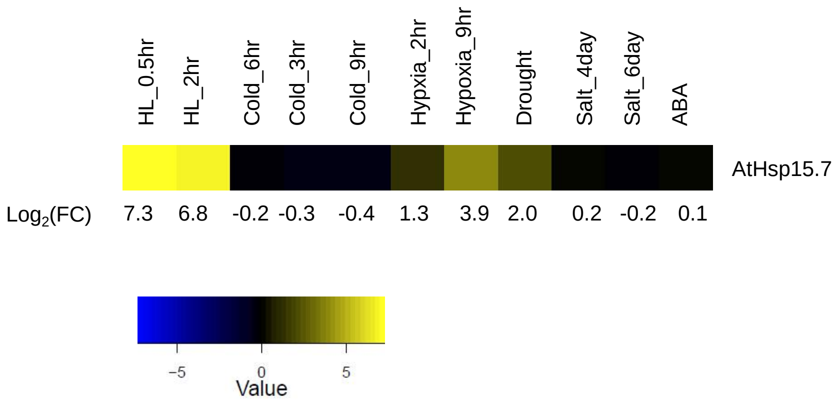

Supplemental Fig A. Expression of the small heat shock protein gene *AtHsp15.7* in response to abiotic stresses. FC, fold change.

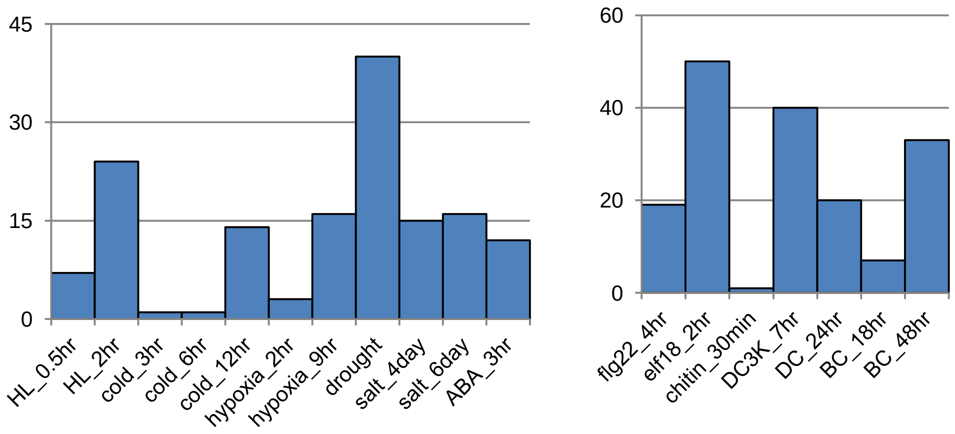

Supplemental Fig B. Total number of peroxisomal genes with significantly changed expression levels in response to stresses. Left, abiotic stresses. Right, biotic stresses. Genes that have log2 normalized fold change >1 or <-1 are considered as significantly regulated.

Table A. Microarray Datasets Used in This Study.

**Abbreviation in heatmap/figure legend**	**Dataset series number**	**Experiment design and description of used dataset**	**Reference or link**
**Developmental**			
Abbreviation in Heatmap	Stage#	Stage description	All data for Developmental stages were downloaded from AtGenExpress: http://arabidopsis.org/servlets/TairObject?type=expression_set&id=1006710873
seedling_cotyledon	1	ATGE_1 development baseline Wt cotyledons 7 days continuous light soil	
seedling_hypocotyl	2	ATGE_2 development baseline Wt hypocotyl 7 days continuous light soil	
seeding_leaves1+2	5	ATGE_5 development baseline Wt leaves 1 + 2 7 days continuous light soil	
adult_leaves	15	ATGE_15 development baseline Wt rosette leaf # 8 17 days continuous light soil	
senescing leaves	25	ATGE_25 development baseline Wt senescing leaves 35 days continuous light soil	
flower	39	ATGE_39 development baseline Wt flowers stage 15 21+ days continuous light soil	
silique_stage3	76	ATGE_76 seed & silique development Wt siliques, w/ seeds stage 3; mid globular to early heart embryos 8 wk long day (16/8) soil	
silique_stage4	77	ATGE_77 seed & silique development Wt siliques, w/ seeds stage 4; early to late heart embryos 8 wk long day (16/8) soil	
silique_stage5	78	ATGE_78 seed & silique development Wt siliques, w/ seeds stage 5; late heart to mid torpedo embryos 8 wk long day (16/8) soil	
seed_stage6	79	ATGE_79 seed & silique development Wt seeds, stage 6, w/o siliques; mid to late torpedo embryos 8 wk long day (16/8) soil	

seed_stage7	81	ATGE_81 seed & silique development Wt seeds, stage 7, w/o siliques; late torpedo to early walking-stick embryos 8 wk long day (16/8) soil	
seed_stage8	82	ATGE_82 seed & silique development Wt seeds, stage 8, w/o siliques; walking-stick to early curled cotyledons embryos 8 wk long day (16/8) soil	
seed_stage9	83	ATGE_83 seed & silique development Wt seeds, stage 9, w/o siliques; curled cotyledons to early green cotyledonsembryos 8 wk long day (16/8) soil	
seed_stage10	84	ATGE_84 seed & silique development Wt seeds, stage 10, w/o siliques; green cotyledons embryos 8 wk long day (16/8) soil	
**abiotic stress**			
HL_0.5hr, HL_2hr	E-MTAB-403	Col-0 plants were grown in growth chambers for 3.5 weeks. Plant leaf tissue was incubated for at least 6 h in a LL growth chamber (light intensity was 65 μmol photons /m^2·s, 22 °C) and then collected as control sample. The leaf tissue was exposed for 0.5 hr and 2 hr to light with an intensity of 1,300 μmol photons /m^2·s, (22 °C).	Jung, H.S., Crisp, P.A., Estavillo, G.M., Cole, B., Hong, F., Mockler, T.C., Pogson, B.J., and Chory, J. (2013). Subset of heat-shock transcription factors required for the early response of Arabidopsis to excess light. Proc Natl Acad Sci USA 110, 14474-14479.
cold_3hr, cold_6hr, cold_24hr	GSE3326	The wild-type seeds were plated on MS agar plates supplemented with 3% sucrose. Seedlings were grown at 22℃ with 16-h-light and 8-h-dark cycles for 2 weeks before being harvested. To avoid variations due to circadian rhythm, all cold treatments were started at 12 PM at 0℃ under light and continued for 0 (untreated control), 3, 6, and 24 h.	Lee, B.H., Henderson, D.A., and Zhu, J.K. (2005). The Arabidopsis cold-responsive transcriptome and its regulation by ICE1. Plant Cell 17, 3155-3175.
hypoxia_2hr, hypoxia_9hr	GSE9719	Seedlings grown in vertical orientation for 7–14 d on solid Murashige–Skoog medium containing 1% sucrose were treated with mixed gases in humidified chambers. Seedlings were deprived of O_2 as well as CO_2 in chambers which were purged with 99.99% Ar(gas), as control. Alternatively,	Sorenson, R., and Bailey-Serres, J. (2014). Selective mRNA sequestration by OLIGOURIDYLATE-BINDING PROTEIN 1 contributes to translational control during hypoxia in Arabidopsis. Proc Natl Acad Sci USA 111, 2373-2378.

		treatment was with 2% O2, 370 ppm CO2, in a balance of N2 for 2hr and 9hr.	
drought	GSE10643	Wild-type plants were grown under normal watering conditions for 24 days and then stressed by completely depriving of irrigation for 10 days.	Zhang, Y., Xu, W., Li, Z., Deng, X.W., Wu, W., and Xue, Y. (2008). F-box protein DOR functions as a novel inhibitory factor for abscisic acid-induced stomatal closure under drought stress in Arabidopsis. Plant Physiol 148, 2121-2133.
salt_4day	GSE53308	Wild type Arabidopsis Col-0 plants were grown hydroponically and treated with or without 150mM NaCl and harvested after 4 days of treatment.	Allu, A.D., Soja, A.M., Wu, A., Szymanski, J., and Balazadeh, S. (2014). Salt stress and senescence: identification of cross-talk regulatory components. J Exp Bot 65, 3993-4008.
salt_6day	GSE16765	Arabidopsis Col-0 were grown in the growth chamber in the absence and presence of salt stress. Plants of 2 weeks were subject to salt treatment for 6 days and were used for RNA extraction.	Chan, Z., Grumet, R., and Loescher, W. (2011). Global gene expression analysis of transgenic, mannitol-producing, and salt-tolerant Arabidopsis thaliana indicates widespread changes in abiotic and biotic stress-related genes. J Exp Bot 62, 4787-4803.
ABA	RIKEN-GODA17&21	Wild-type seedlings were treated with ABA and mock for 3 hr.	Nemhauser, J.L., Hong, F., and Chory, J. (2006). Different plant hormones regulate similar processes through largely nonoverlapping transcriptional responses. Cell *126*, 467-475. http://arabidopsis.org/servlets/TairObject?type=hyb_descr_collection&id=1007964750
**biotic stress**			
flg22_4hr	GSE11807	Six-week old Col-0 plants were infiltrated with 1 µM flg22 or ddH2O and harvested 4 hours later. Total RNA was extracted, biotinlabeled and hybridized to the Affymetrix ATH1 chip. Gene expression values from treated sample were compared to that of mock-treated sample	Bethke G, Unthan T, Uhrig JF, Poschl Y, Gust AA, et al. (2009) Flg22 regulates the release of an ethylene response factor substrate from MAP kinase 6 in Arabidopsis thaliana via ethylene signaling. Proc Natl Acad Sci USA 106: 8067-8072.

elf18_2hr	GSE34047	3-week old Col-0 plants were infiltrated with 10 µM elf18 or mock and harvested after 2 hrs. Three biological replicates were included	Pajerowska-Mukhtar KM, Wang W, Tada Y, Oka N, Tucker CL, et al. (2012) The HSF-like transcription factor TBF1 is a major molecular switch for plant growth-to-defense transition. Curr Biol 22: 103-112.
chitin_30min	GSE28227	2-week old MS medium-grown seedlings were treated with chitooctaose at a final concentration of 1 µM for 30 minutes. The controls were similarly treated with an equivalent amount of ddH2O. Seedlings were harvested for RNA isolation. Three biological replicates were conducted for the experiment	http://www.ncbi.nlm.nih.gov/geo/query/acc.cgi?acc=GSE28227
DC3K_7hr; DC3K_24hr	GSE5520	4-week old Arabidopsis leaves were treated with bacterial pathogen Pseudomonas syringae pv. Tomato DC3000. The first leaf sample was treated with bacteria at the concentration of 10*8 bacteria/ml and sampled 7 hours later, and the second leaf sample was treated with bacteria at the concentration of 10*6 bacteria/ml and sampled 24 hours later	Thilmony R, Underwood W, He SY (2006) Genome-wide transcriptional analysis of the Arabidopsis thaliana interaction with the plant pathogen Pseudomonas syringae pv. tomato DC3000 and the human pathogen Escherichia coli O157:H7. Plant J 46: 34-53.
BC_18hr; BC_48hr	GSE5684	Adult Col-0 leaves were inoculated by placing 4 5-µl drops of 5x10*5 Botrytis cinerea spore solution. Control leaves were spotted with droplets of 24g/L potato dextrose broth medium. Samples were collected at 18 hrs and 48 hrs after inoculation.	http://www.ncbi.nlm.nih.gov/geo/query/acc.cgi?acc=GSE5684

Table B. Arabidopsis Peroxisomal Gene List.

160 peroxisomal genes have expression data and were used for co-expression analysis. The last eight genes do not have microarray probes.

Order	Gene name	A.t Locus	Microarray probe ID	Annotation
1	*ACH2*	AT1G01710	261560_at	acyl-CoA thioesterase family protein
2	*PEX11c*	AT1G01820	261534_at	PEX11c, peroxisome elongation
3	*PEX6*	AT1G03000	263170_at	peroxin6
4	*st4*	AT1G04290	263661_at	thioesterase family protein
5	*KAT1*	AT1G04710	264608_at	3-keto-acyl-CoA thiolase 1
6	*ACX3*	AT1G06290	260789_s_at	acyl-CoA oxidase 3
7	*ACX6*	AT1G06310	259419_at	acyl-CoA oxidase 6
8	*ACD31.2*	AT1G06460	262629_at	alpha-crystallin domain 31.2
9	*NDA1*	AT1G07180	256057_at	alternative NAD(P)H dehydrogenase 1
10	*GLX1*	AT1G11840	264372_at	glyoxalase i homolog 1
11	*GAPC2*	AT1G13440	259361_at	glyceraldehyde-3-phosphate dehydrogenase
12	*UP6*	AT1G16730	255763_at	unknown protein 6
13	*DHAR*	AT1G19570	261149_s_at	dehydroascorbate reductase 1
14	*4Cl3*	AT1G20480	259569_at	4-coumaroyl-CoA synthase family protein
15	*OPCL1*	AT1G20510	259518_at	opc-8:0 -CoA ligase1
16	*AAE1*	AT1G20560	259545_at	acyl-activating enzyme 1
17	*CAT3*	AT1G20620	259544_at	catalase 3
18	*CAT1*	AT1G20630	259517_at	catalase 1
19	*ATF1*	AT1G21770	262499_at	acyl-CoA n-acyltransferases
20	*GGT1*	AT1G23310	262988_at	alanine-2-oxoglutarate aminotransferase 1
21	*DEG15*	AT1G28320	245687_at	endopeptidase
22	*UP9*	AT1G29120	260889_at	unknown protein 9
23	*PEX7*	AT1G29260	260844_at	peroxin 7

24	*AAE14*	AT1G30520	261801_at	acyl-activating enzyme 14
25	*PEX11a*	AT1G47750	261739_at	PEX11a, peroxisome elongation
26	*st1*	AT1G48320	262237_at	thioesterase family protein
27	*pxPfkB*	AT1G49350	262398_at	pfkb-type carbohydrate kinase family protein
28	*NQR*	AT1G49670	261601_at	involved in oxidative stress tolerance.
29	*ICDH*	AT1G54340	262962_at	isocitrate dehydrogenase
30	*NS*	AT1G60550	264920_at	naphthoate synthase
31	*ECI*	AT1G65520	264627_at	enoyl-CoA hydratase/isomerase family protein
32	*PAO4*	AT1G65840	262933_at	polyamine oxidase 4
33	*BZO1*	AT1G65880	261915_at	benzoate-CoA ligase
34	*AAE12*	AT1G65890	261922_at	acyl-activating enzyme 12
35	*HPR1*	AT1G68010	260014_at	hydroxypyruvate reductase 1
36	*GGT2*	AT1G70580	260309_at	glutamate:glyoxylate aminotransferase 2
37	*ECH2*	AT1G76150	261771_at	monofunctional enoyl-CoA hydratase 2
38	*ATF2*	AT1G77540	259706_at	histone acetyltransferase
39	*PEX2*	AT1G79810	261348_at	peroxin2
40	*SMP2*	AT2G02510	267239_at	NADH dehydrogenase
41	*OPR3*	AT2G06050	265530_at	OPDA-reductase 3
42	*AGT1*	AT2G13360	263350_at	alanine:glyoxylate aminotransferase
43	*DRP3B*	AT2G14120	263278_at	dynamin-related protein 3B
44	*MDH1*	AT2G22780	266457_at	peroxisomal NAD-malate dehydrogenase 1
45	*SOX*	AT2G24580	263788_at	sarcosine oxidase family protein
46	*Uri*	AT2G26230	267374_at	uricase/urate oxidase putative
47	*PEX10*	AT2G26350	267433_at	peroxin 10
48	*CoAE*	AT2G27490	265637_at	dephospho-CoA kinase
49	*st5*	AT2G29590	266298_at	thioesterase family protein
50	*NDA2*	AT2G29990	266835_at	alternative NAD(P)H dehydrogenase 2

51	*CHYH1*	AT2G30650	267571_at	3-hydroxyisobutyryl-coenzyme a hydrolase
52	*CHYH2*	AT2G30660	267572_at	3-hydroxyisobutyryl-coenzyme a hydrolase
53	*UP3*	AT2G31670	263449_at	unknown protein 3
54	*KAT2*	AT2G33150	245168_at	3-keto-acyl-CoA thiolase 2
55	*ACX5*	AT2G35690	265843_at	acyl-CoA oxidase 5
56	*GLH*	AT2G38180	267096_at	gdsl-motif lipase/hydrolase family protein
57	*PXN/PMP38/PMP36*	AT2G39970	267363_at	peroxisomal membrane protein 36
58	*PM16*	AT2G41790	260554_at	peptidase m16 family protein
59	*CuAO*	AT2G42490	265882_at	copper amine oxidase
60	*CSY3*	AT2G42790	263986_at	citrate synthase 3
61	*PAO2*	AT2G43020	265244_at	polyamine oxidase 2
62	*PEN2*	AT2G44490	267392_at	o-glycosyl compounds hydrolase
63	*PEX16*	AT2G45690	267512_at	peroxin 6
64	*PEX11d*	AT2G45740	266925_at	PEX11d, peroxisome elongation
65	*SO*	AT3G01910	258948_at	sulfite oxidase
66	*SDRc*	AT3G01980	258976_at	short-chain dehydrogenase/reductase (sdr) family protein
67	*6PGDH*	AT3G02360	256328_at	6-phosphogluconate dehydrogenase family protein
68	*PEX19A*	AT3G03490	259052_at	peroxin 19-1
69	*PEX12*	AT3G04460	258627_at	peroxin 22
70	*PNC1*	AT3G05290	259306_at	peroxisomal adenine nucleotide carrier 1
71	*LACS6*	AT3G05970	258563_at	long-chain acyl-CoA synthetase 6
72	*IBR3*	AT3G06810	258525_at	acyl-CoA dehydrogenase
73	*MFP2*	AT3G06860	258555_at	multifunctional protein 2
74	*CML3*	AT3G07490	259064_at	calcium ion binding
75	*PEX13*	AT3G07560	259068_at	peroxin 13
76	*SDRb*	AT3G12800	257687_at	short-chain dehydrogenase
77	*HAOX1/2*	AT3G14130, AT3G14150	257004_s_at	putative glycolate oxidase

78	*GOX1/2*	AT3G14415, AT3G14420	258359_s_at	glycolate oxidase
79	*HBCDH*	AT3G15290	257052_at	3-hydroxybutyryl-CoA dehydrogenase, putative
80	*AAE7*	AT3G16910	257880_at	acyl-activating enzyme 7
81	*GPK1*	AT3G17420	257295_at	glyoxysomal protein kinase 1
82	*PEX3A*	AT3G18160	258150_at	peroxin 3a
83	*SCO3*	AT3G19570	257047_at	snowy cotyledon3
84	*DRP5B*	AT3G19720	257045_at	dynamin-related protein 5B
85	*ICL*	AT3G21720	257947_at	isocitrate lyase
86	*PEX22*	AT3G21865	257953_at	peroxin 22
87	*GR1*	AT3G24170	257252_at	glutathione-disulfide reductase 1
88	*MDAR4*	AT3G27820	257227_at	monodehydroascorbate reductase 4
89	*PEX11b*	AT3G47430	252411_at	PEX11b, peroxisome elongation
90	*B12D1*	AT3G48140	252348_at	senescence-associated protein
91	*BADH*	AT3G48170	252354_at	betaine aldehyde dehydrogenase
92	*MIF*	AT3G51660	252076_at	macrophage migration inhibitory factor family protein
93	*ACX4*	AT3G51840	246304_at	acyl-CoA oxidase 4
94	*MDAR1*	AT3G52880	252024_at	monodehydroascorbate reductase
95	*SDRd*	AT3G55290	251780_s_at	short-chain dehydrogenase/reductase (sdr) family protein]
96	*CDC*	AT3G55640	251757_at	mitochondrial substrate carrier family protein
97	*ZnDH*	AT3G56460	251687_at	zinc-binding dehydrogenase
98	*HIT3*	AT3G56490	251707_at	histidine triad family protein
99	*FIS1A*	AT3G57090	251659_at	fission protein 1A
100	*CP*	AT3G57810	251558_at	OTU-like cysteine protease
101	*CSY1*	AT3G58740	251540_at	citrate synthase 1
102	*CSY2*	AT3G58750	251541_at	citrate synthase 2
103	*PMD1*	AT3G58840	251556_at	peroxisome and mitochodria division protein
104	*PAO3*	AT3G59050	251505_at	polyamine oxidase 3

105	*PEX11e*	AT3G61070	251352_at	PEX11e, peroxisome elongation
106	*st3*	AT3G61200	251307_at	thioesterase family protein
107	*ACH*	AT4G00520	255679_at	acyl-CoA thioesterase family protein
108	*EH3*	AT4G02340	255525_at	putative epoxide hydrolase
109	*MCD*	AT4G04320	255327_at	malonyl-CoA decarboxylase family protein
110	*PMP22*	AT4G04470	255338_at	peroxisomal membrane protein 22
111	*4CL1*	AT4G05160	255263_at	putative 4-coumaroyl-CoA synthase
112	*IBR1*	AT4G05530	255240_at	short-chain dehydrogenase
113	*NDPK1*	AT4G09320	255089_at	nucleoside diphosphate kinase 1
114	*SCPL20*	AT4G12910	254791_at	serine carboxypeptidase-like 20
115	*IBR10*	AT4G14430	245359_at	enoyl-CoA hydratase/isomerase family protein
116	*ECHIA*	AT4G16210	245484_at	enoyl-CoA hydratase/isomerase family protein
117	*HIT1*	AT4G16566	245337_at	histidine triad family protein
118	*ACX1*	AT4G16760	245249_at	acyl-CoA oxidase 1
119	*GOX3*	AT4G18360	254630_at	putative glycolate oxidase
120	*4Cl5*	AT4G19010	254600_at	4-coumaroyl-coa synthase family protein
121	*NDB1*	AT4G28220	253810_at	NAD(P)H dehydrogenase b
122	*AIM1*	AT4G29010	253759_at	abnormal inflorescence meristem1 , enoyl-CoA hydratase
123	*DRP3A*	AT4G33650	253306_at	dynamin-related protein 3A
124	*APX3*	AT4G35000	253223_at	ascorbate peroxidase 3
125	*CAT2*	AT4G35090	253174_at	catalase 2
126	*RDL1*	AT4G36880	246250_at	cysteine-type peptidase
127	*AGT2*	AT4G39660	252855_at	alanine:glyoxylate aminotransferase 2
128	*PXA1/CTS*	AT4G39850	252830_at	peroxisomal ABC transporter 1
129	*MLS*	AT5G03860	250868_at	malate synthase
130	*BIOTIN_F*	AT5G04620	250837_at	7-keto-8-aminopelargonic acid synthase
131	*CPK1*	AT5G04870	246955_at	calcium-dependent protein kinase isoform

132	*PEX1*	AT5G08470	250520_at	peroxin 1, ATPase
133	*MDH2*	AT5G09660	250498_at	peroxisomal NAD-malate dehydrogenase 2
134	*ASP3*	AT5G11520	250385_at	aspartate aminotransferase 3
135	*ELT1*	AT5G11910	250299_at	esterase/lipase/thioesterase family protein
136	*FIS1B*	AT5G12390	245178_at	mitochodria and peroxisome fission protein
137	*AAE5*	AT5G16370	250114_s_at	acyl-activating enzyme 5
138	*ATMS1*	AT5G17920	259343_s_at	methionine synthesis 1
139	*CSD3*	AT5G18100	250016_at	copper superoxide dismutase 3
140	*NUDT19*	AT5G20070	246126_at	nudix hydrolase homolog 19
141	*AAE17*	AT5G23050	249869_at	acyl-activating enzyme 17
142	*6PGL*	AT5G24400	249733_at	6-phosphoglucunolactonase
143	*PEX4*	AT5G25760	246862_at	peroxin4, ubiquitin-protein ligase
144	*PNC2*	AT5G27520	246779_at	peroxisomal adenine nucleotide carrier 2
145	*LACS7*	AT5G27600	246789_at	long-chain acyl-CoA synthetase 7
146	*AtHsp15.7*	AT5G37670	249575_at	15.7 kda class i-related small heat shock protein-like
147	*GSTT1*	AT5G41210	249291_at	glutathione s-transferase (class theta) 1
148	*SCP2*	AT5G42890	249178_at	sterol carrier protein 2
149	*AtDCI*	AT5G43280	249145_at	delta(3,5),delta(2,4)-dienoyl-CoA isomerase 1
150	*UP5*	AT5G44250	249064_at	unknown protein 5
151	*LON2*	AT5G47040	248818_at	lon protease homolog 2
152	*ACAT1.3*	AT5G47720	248779_at	putative acetyl-CoA c-acyltransferase
153	*ACAT2*	AT5G48230	248690_at	acetoacetyl-CoA thiolase 2
154	*KAT5*	AT5G48880	248625_at	3-keto-acyl-CoA thiolase 5
155	*PEX5*	AT5G56290	248010_at	peroxin 5, peroxisome matrix targeting signal-1 binding protein
156	*TLP*	AT5G58220	247858_at	transthyretin-like protein
157	*PEX14*	AT5G62810	247422_at	peroxin 14
158	*4CL2*	AT5G63380	247380_at	4-coumarate-CoA ligase family protein

159	*ACX2*	AT5G65110	247176_at	acyl-CoA oxidase 2
160	*CHY1*	AT5G65940	247117_at	beta-hydroxyisobutyryl-CoA hydrolase 1
161	*AAE18*	AT1G48635	no probe	acyl-activating enzyme 18
162	*PEX3B*	AT1G50510	no probe	peroxin 3B
163	*IndA*	AT1G55320	no probe	indigoidine synthase a family protein
164	*NADK3*	AT1G78590	no probe	NADH kinase
165	*APEM9*	AT3G10572	no probe	required for both pts1- and pts2-dependent protein transport
166	*PEX19B*	AT5G17550	no probe	peroxin 19B
167	*MIA40*	AT5G23395	no probe	mitochondrial intermembrane space assembly machinery 40
168	*HIT2*	AT5G48545	no probe	histidine triad nucleotide-binding 3

Table C. Mutants Used in the Primary Screen for Drought Tolerance.

KO, knock-out; KD, knock-down。

Mutant	SALK_ID	Gene expression	Transcriptional regulation by drought, $\log_2$(FC)	Reference
aba1	SALK_059469	KO	Control	[1]
hpr1-1	SALK_067724	KO	limited up-regulation, 0.44	[2]
hpr1-2	SALK_143584	KO		
cat2	SALK_076998	KO	up-regulated by drought, 1.70	[3]
gox1	SAIL_177_G11	KO	limited up-regulation by drought, 0.42	[4]
gox3	SALK_020909	KO	up-regulated by drought, 1.10	[5]
hsp15.7-1	SALK_038951	KD	up-regulated by drought, 2.05	[6]
hsp15.7-2	SALK_107711	KO		
pao2-1	SALK_046281	KO	down-regulated by drought, -1.37	[7]
pao2-2	SALK_062035			
pxn-1	SALK_038951	KO	limited up-regulation by drought, 0.03	[8]
pxn-2	SALK_107711	KD		
csy3	SALK_076319	KO	up-regulated by drought, 2.23	
mif	SALK_037373		up-regulated by drought, 1.79	
lon2-2	SALK_043857	KO	up-regulated by ABA/drought, 1.98/0.51	[9]
kat5-1	SALK_132871		down-regulated by drought, -1.93	
kat5-2	SALK_144464	KD		[10]
acx3-1	SALK_128947		limited up-regulation by drought, 0.05	
acx3-2	SALK_044956	KO		[11]
acx4-1	SALK_000879	KO	up-regulated by drought, 1.21	[11]
acx4-2	SALK_065013	KO		
acx6	SALK_023093	KO	limited up-regulation by drought, 0.01	[11]

acx1/5	SALK_041464/SALK_009998	KO	up-regulated by drought, 1.38/0.51	[12]
aae14	SALK_038308		down-regulated by drought, -2.27	
chy1-1	SALK_025417	KO	limited up-regulation by drought, 0.82	[13]
chy1-2	SALK_102725			

References

1. Riboni M, Galbiati M, Tonelli C, Conti L (2013) GIGANTEA enables drought escape response via abscisic acid-dependent activation of the florigens and SUPPRESSOR OF OVEREXPRESSION OF CONSTANS. Plant Physiol 162: 1706-1719.
2. Timm S, Nunes-Nesi A, Parnik T, Morgenthal K, Wienkoop S, et al. (2008) A cytosolic pathway for the conversion of hydroxypyruvate to glycerate during photorespiration in Arabidopsis. Plant Cell 20: 2848-2859.
3. Shibata M, Oikawa K, Yoshimoto K, Kondo M, Mano S, et al. (2013) Highly oxidized peroxisomes are selectively degraded via autophagy in Arabidopsis. Plant Cell 25: 4967-4983.
4. Rojas CM, Senthil-Kumar M, Wang K, Ryu CM, Kaundal A, et al. (2012) Glycolate oxidase modulates reactive oxygen species-mediated signal transduction during nonhost resistance in Nicotiana benthamiana and Arabidopsis. Plant Cell 24: 336-352.
5. Quan S, Yang P, Cassin-Ross G, Kaur N, Switzenberg R, et al. (2013) Proteome analysis of peroxisomes from etiolated Arabidopsis seedlings identifies a peroxisomal protease involved in beta-oxidation and development. Plant Physiol 163: 1518-1538.
6. Ma C (2005) Subcellular and functional analyses of two small heat shock proteins and protein kinases from peroxisomes of Arabidopsis thaliana L. PhD Dissertatoin.
7. Kim DW, Watanabe K, Murayama C, Izawa S, Niitsu M, et al. (2014) Polyamine Oxidase5 Regulates Arabidopsis Growth through Thermospermine Oxidase Activity. Plant Physiol 165: 1575-1590.
8. Bernhardt K, Wilkinson S, Weber AP, Linka N (2012) A peroxisomal carrier delivers NAD(+) and contributes to optimal fatty acid degradation during storage oil mobilization. Plant J 69: 1-13.
9. Lingard MJ, Bartel B (2009) Arabidopsis LON2 is necessary for peroxisomal function and sustained matrix protein import. Plant Physiol 151: 1354-1365.
10. Wiszniewski A (2011) Dissecting functions of members of β-oxidation multi-gene families in Arabidopsis. PhD Dissertatoin.
11. Adham AR, Zolman BK, Millius A, Bartel B (2005) Mutations in Arabidopsis acyl-CoA oxidase genes reveal distinct and overlapping roles in beta-oxidation. Plant J 41: 859-874.
12. Schilmiller AL, Koo AJ, Howe GA (2007) Functional diversification of acyl-coenzyme A oxidases in jasmonic acid biosynthesis and action. Plant Physiol 143: 812-824.

13. Ibdah M, Pichersky E (2009) Arabidopsis Chy1 null mutants are deficient in benzoic acid-containing glucosinolates in the seeds. Plant Biol (Stuttg) 11: 574-581.
